# A retrospective study of survival and risk factors for mortality among people living with HIV who received antiretroviral treatment in a resource-limited setting

**DOI:** 10.1186/s12981-021-00397-1

**Published:** 2021-10-12

**Authors:** Weerawat Manosuthi, Lantharita Charoenpong, Chalor Santiwarangkana

**Affiliations:** 1 Present Address: Bamrasnaradura Infectious Diseases Institute, Department of Diseases Control, Ministry of Public Health, Nonthaburi, 11000 Thailand; 2 National Health Security Office Region 4, Saraburi, 18000 Thailand

**Keywords:** HIV, Survival, Thailand

## Abstract

**Background:**

The availability and accessibility of effective antiretroviral therapy (ART) for people living with HIV (PLWH) has substantially improved in the past two decades in resource-limited settings. Therefore, evaluation of survival is needed in the current setting.

**Method:**

We retrospectively analyzed secondary data of the national AIDS program database from national health security region number 4 among PLWH who were ART-naive between January 2014 and December 2018. All PLWH were followed until December 2019 to evaluate their survival status and possible risk factors related to death.

**Results:**

A total of 42,229 PLWH were identified, of which 14,053 were ART-naive and thus enrolled in the study. Sixty-seven percent were male, the mean ± SD age was 35 ± 12 years, and the median (IQR) baseline CD4 count was 162 (44–353) cells/mm^3^. Regarding medical care benefits, 46% had a universal health coverage scheme, 34% had a national social security scheme, and 2% had a civil servants medical benefit scheme. A total of 2142 (15%) mortalities occurred during the total follow-up period of 28,254 patient-years. The mortality rate was 7.5 (95% CI 7.2–7.9) per 100 person-years. Survival rates at 1, 2, 3, 4 and 5 years after HIV registration were 88.2% (95% CI 87.6–88.7%), 85.3% (95% CI 84.6–85.9%), 82.9% (95% CI 81.9–83.4%), 81.3% (95% CI 80.5–82.0%) and 75.1% (95% CI 73.5–76.8%), respectively. The Cox proportional hazards model showed that all-cause mortality was associated with a history of ART switching (HR = 7.06, 95% CI 4.53–11.00), major opportunistic infections during ART (HR = 1.93, 95% CI 1.35–2.77), baseline CD4 count ≤ 200 vs. > 500 cells/mm3 (HR = 4.00, 95% CI 1.45–11.11), age ≥ 50 vs. < 30 years (HR = 1.77, 95% CI 1.12–2.78), and receiving nevirapine-based regimens(HR = 1.43, 95% CI 1.04–1.97).

**Conclusions:**

This study demonstrated the substantial mortality rate over the consecutive 5 years of the follow-up period among PLWH who received ART in a resource-limited setting. Early case finding and prompt initiation of ART as well as continuous HIV care are a cornerstone to improve survival.

## Introduction

Currently, there is an estimated 37.7 million people living with HIV (PLWH) globally [1]. In Thailand, there is an estimated 500,000 PLWH, and 12,000 people died of AIDS-related illnesses in 2020. As such, Thailand is considered to be one of the countries with a high HIV burden in Asia and the Pacific regions, accounting for 9% of the region’s total number of PLWH [1]. The national strategy to end AIDS during 2017–2030 has set the targets of reducing new cases of HIV to below 1000 cases per year, reducing AIDS mortality to no greater than 4000 cases per year, and decreasing HIV stigma and discrimination by 90% [2]. In 2020, Joint United Nations Programme on HIV/AIDS (UNAIDS)’ 90–90–90 targets in Thailand were 94% of PLWH diagnosed and knew their status, 83% received ART, and of those on treatment, 97% were virologically suppressed [1]. HIV suppression rates among PLWH have increased significantly, up to 77% in 2020, compared to only 53% in 2015 [3].

Access to ART in Thailand has continuously increased, resulting in enormous progress with virus suppression to achieve the third 90 target of UNAIDS in recent years, and the estimated number of AIDS-related deaths has gradually declined, but it is still more than 10,000 cases per year [2]. Studies on mortality and risk factors for PLWH in Thailand are lacking. In addition, real-world data regarding long-term survival and associated factors are needed to inform clinical care, guide policy, and help evaluate program effectiveness. Therefore, this study aimed to investigate the survival rate and possible risk factors related to death among PLWH in whom ART use is implemented nationwide.

## Materials and methods

A retrospective data review study was conducted among PLWH who were registered in the national AIDS program database at national health security office region 4, which comprised eight provinces, including the Nonthaburi, Lopburi, Singhburi, Saraburi, Ayutthaya, Angthong, Nakhonnayok, and Pathumthani provinces. All 12 health districts in the country are responsible for the approximately 3–6 million people living in the provinces of each health district [4]. The study was reviewed and approved by the institutional review board of Bamrasnaradura Infectious Diseases Institute (S021 h/62). The institution’s institutional review board waived the requirement to obtain informed consent. The confidentiality and privacy of the information were assured by removing the identity of the individual PLWH and replacing it with a new anonymous code given by the national health security office. The inclusion criteria of the study were as follows: (1) PLWH > 15 years of age, (2) registered in the national HIV program database region 4, (3) antiretroviral-naive treatment, and (4) initiated ART between January 2014 and December 2018. The exclusion criteria were as follows: (1) no data available after registration, and (2) no follow-up visit after registration. All patients were followed up for vital status until December 2019. Each PLWH was classified into the following two groups based on their vital status during the follow-up period: (1) surviving group, and (2) nonsurviving group.

The primary outcome of interest was time from HIV registration in the database to death, which was calculated by subtracting the date of death from the date on which the patient was registered. The patients were censored at the date of the last visit if they were lost to follow-up or censored at the date of referral. The patients’ vital status in this national HIV database was checked to differentiate between those who lost to follow-up and those who had died. The secondary objectives were to determine the possible risk factors related to death. Possible risk factors along with other data collected were studied. These included patients’ demographics, i.e., sex measured as male or female, baseline CD4 cell count upon registration, history of major opportunistic infections (OIs) prior registration measured as yes or no, major OIs during the follow-up period measured as yes or no, major opportunistic infection was classified according to WHO clinical stage IV [5], medical care benefit was classified as universal coverage (UC) or social security scheme (SS) or civil servant medical benefit scheme (CSMBS), ART regimen as measure as efavirenz-containing or nevirapine-containing or lopinavir/ritonavir-containing, time to achieving undetectable HIV-RNA viral load form registration, history of ART switching due to any reason measured as yes or no, and time since HIV registration in the database to start ART. Data cleaning and analysis were delegated to the Medical Research Network of the Consortium of Thai Medical Schools (MedResNet). The report of the present study followed the strengthening of the reporting of studies conducted using observational, routinely collected data (RECORD) guidelines, which is a reporting guideline for studies conducted using routinely collected health data, such as health administrative data, electronic medical record data, primary care surveillance data, and disease registries [6].

## Statistical analysis

The mean ± standard deviation (SD) used when the numerical data were normally distributed while the median (interquartile range 25th and 75th, IQR) was used when skewed and frequencies (%) are used to describe PLWH characteristics in each group. The chi-squared test and Mann–Whitney *U* test were used to compare categorical and continuous variables between the two groups, respectively. The Kaplan–Meier method was used to estimate the overall survival and the median time to death after HIV registration in the national HIV program database. The log-rank test was used to compare the median time to death among groups. The present study applied Cox regression for all explanatory factors effecting and adjusted all factors affected on the survival rate. Cox proportional hazards model was used to determine the chance of death after HIV registration by adjusting for confounding factors, such as sex, previous OIs, baseline CD4 cell count, medical care benefit upon national AIDS program database registration, history of ART switching due to any reason and initial ART regimen. The hazard ratio (HR) and its 95% confidence interval (CI) were estimated. All analyses were performed using STATA version 15.1 (College Station, Texas 77845, USA). A *p* value less than 0.05 was considered statistically significant.

## Results

**Fig. 1:**
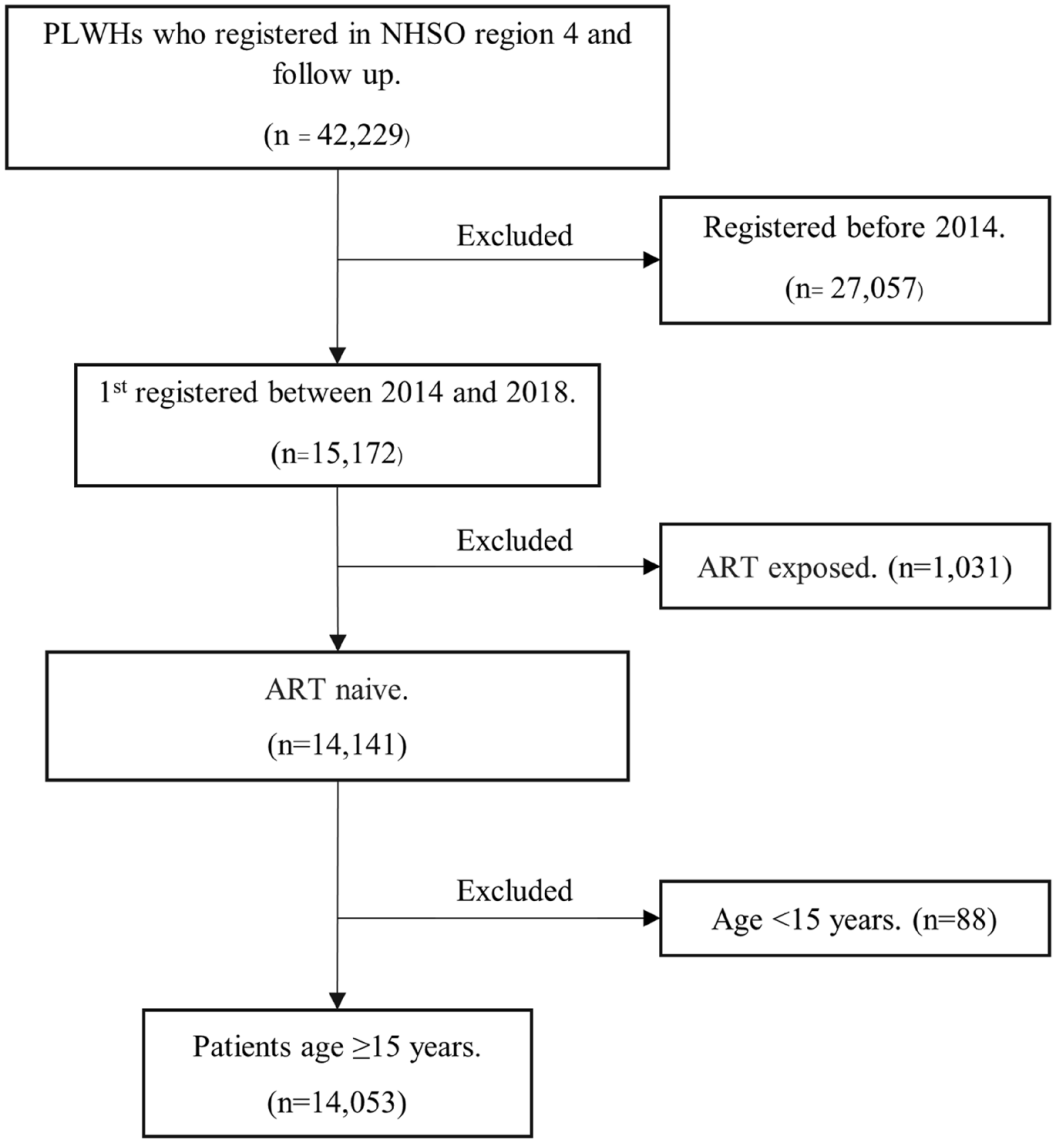
Schematic of study enrollment

Based upon data from the national AIDS program database, a total of 42,229 PLWH were identified of which 14,053 were ART naive and enrolled in the study. Figure 1 shows the schematic of study enrollment. The median (IQR) time of the follow-up period was 1.76 (0.66–3.23) years. The characteristics of PLWH were described and compared between the two study groups, as shown in Table 1. Of 14,053 PLWH, 67% were male, and the mean ± SD age was 35 ± 12 years. In terms of medical care benefits, 64%, 34%, and 2% of PLWH were covered under a universal health coverage scheme, a national social security scheme, and a civil servants medical benefit scheme, respectively. The overall median (IQR) baseline CD4 count was 162 (44–353) cells/mm^3^. The patients in the nonsurviving group had a higher proportion of older age, history of OIs before HIV registration, OIs during the follow-up period, received nevirapine-based ART regimen, lower baseline CD4 cell count, switching ART regimen during follow-up period than the patients in the surviving group (*p* < 0.05).

**Table 1:** Baseline demographic data between the two groups

Factors	Surviving groupv n = 11,911	Nonsurviving group n = 2142	Total n = 14,053	*p* value
	Number (%)	Number (%)	Number (%)	
Sex				0.385
Male	8060 (67.7%)	1429 (66.7%)	9489 (67.5%)	
Female	3851 (32.3%)	713 (33.3%)	4564 (32.5%)	
Age range, years				< 0.001
< 30	4671 (39.2%)	378 (17.6%)	5049 (35.9%)	
30–49	5976 (50.2%)	1267 (59.2%)	7243 (51.5%)	
≥ 50	1264 (10.6%)	497 (23.2%)	1761 (12.5%)	
Mean ± SD	34 ± 11	41 ± 12	35 ± 12	
Medical benefit schemes				< 0.001
UC	7321 (62.1%)	1543 (72.8%)	8864 (63.7%)	
SS	4199 (35.6%)	540 (25.5%)	4739 (34.1%)	
CSMBS	271 (2.3%)	36 (1.7%)	307 (2.2%)	
Total	11,791 (100%)	2119 (100%)	13,910 (100%)	
History of previous OIs prior to ART				< 0.001
No	10,957 (92.0%)	1801 (84.1%)	12,758 (90.8%)	
Yes	954 (8.0%)	341 (15.9%)	1295 (9.2%)	
OIs during ART				< 0.001
No	10,995 (92.3%)	1870 (87.3%)	12,865 (91.5%)	
Yes	916 (7.7%)	272 (12.7%)	1188 (8.5%)	
ART regimens				< 0.001
Efavirenz-containing	7438 (82.6%)	713 (76.9%)	8151 (82.1%)	
Nevirapine-containing	1238 (13.7%)	197 (21.3%)	1435 (14.4%)	
Lopinavir/ritonavir-containing	328 (3.6%)	17 (1.8%)	345 (3.5%)	
Total	9004 (100.0%)	927 (100.0%)	9931 (100.0%)	
Baseline CD4 count, cells/mm^3^				< 0.001
≤200	3514 (52.1%)	690 (80.5%)	4204 (55.3%)	
201–500	2385 (35.4%)	135 (15.7%)	2520 (33.2%)	
> 500	846 (12.5%)	32 (11.6%)	878 (11.5)	
Total	6745 (100%)	857 (100.0%)	7602 (100.0%)	
Median (IQR25th–75th)	186 (51–367)	50 (19–145)	162 (44–353)	
Time since registration to start ART, years				0.305
Median (IQR25th–75th)	0.06 (0.02–0.13)	0.08 (0.04–0.16)	0.06 (0.02–0.13)	
ART switch during follow-up period				< 0.001
No	6027 (64.4%)	34 (14.2%)	6061 (63.1%)	
Yes	3332 (35.6%)	206 (85.8%)	3538 (36.9%)	
Total	9359 (100.0%)	240 (100.0%)	9599 (100.0%)	
Reasons to switch ART				0.049
Complication	1733 (52.0%)	98 (47.6%)	1831 (51.8%)	
Treatment failure	214 (6.4%)	22 (10.7%)	236 (6.7%)	
Data not available	1385 (41.6%)	86 (41.7%)	1471 (41.6%)	
Total	3332 (100.0%)	206 (100.0%)	3538 (100.0%)	
Time to plasma HIV viral load < 40 copies/mL, years				
Mean ± SD	1.4 ± 0.5	1.4 ± 0.6	1.4 ± 0.5	0.811

**Fig. 2:**
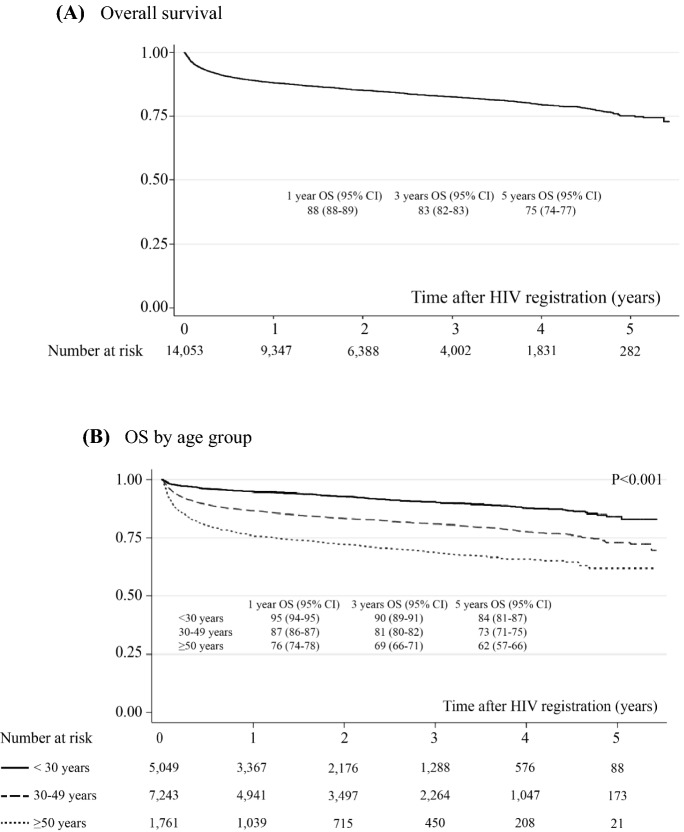

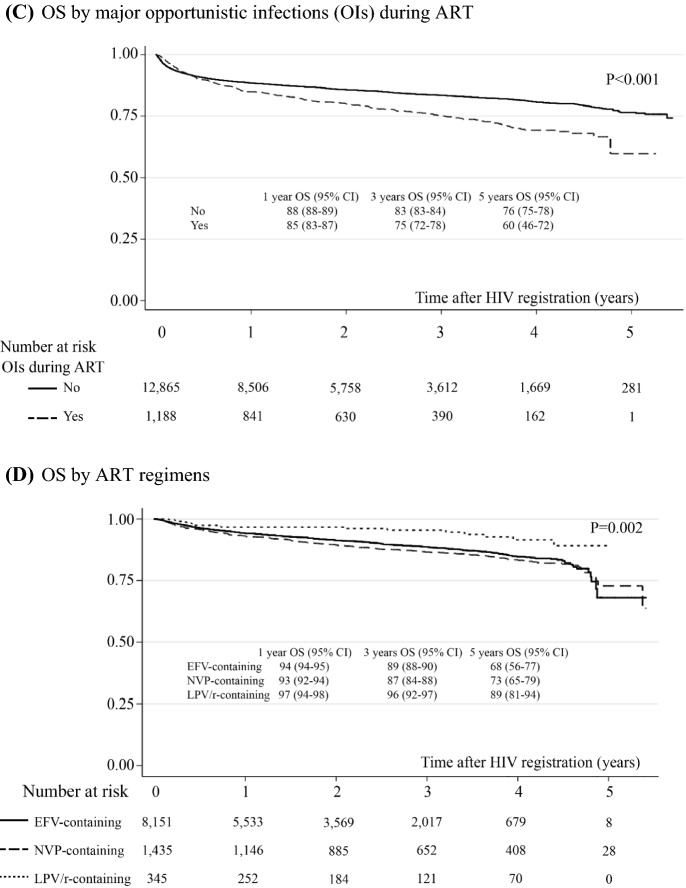

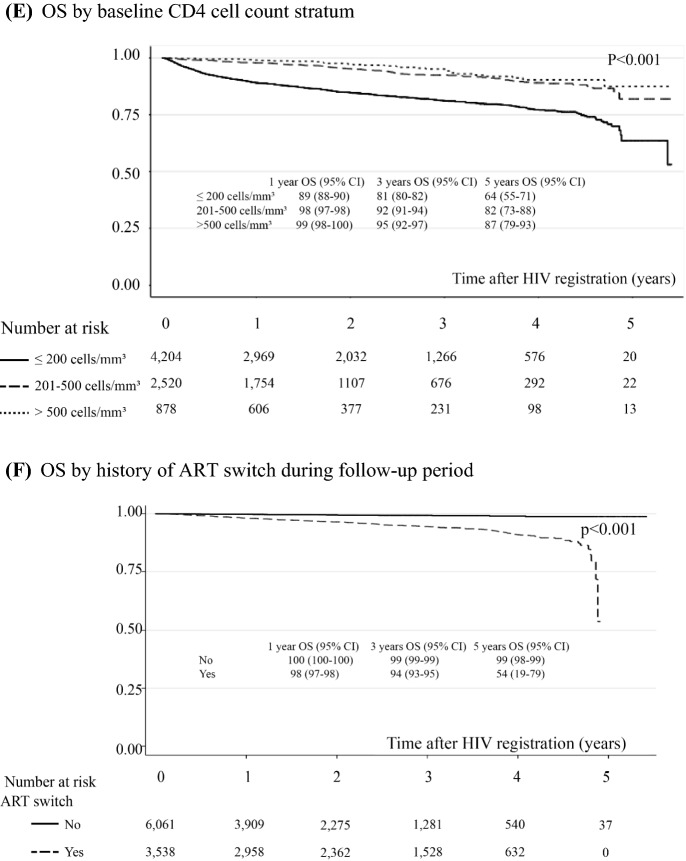
Overall survival (OS) probability over 5-year study period

**Fig. 3:**
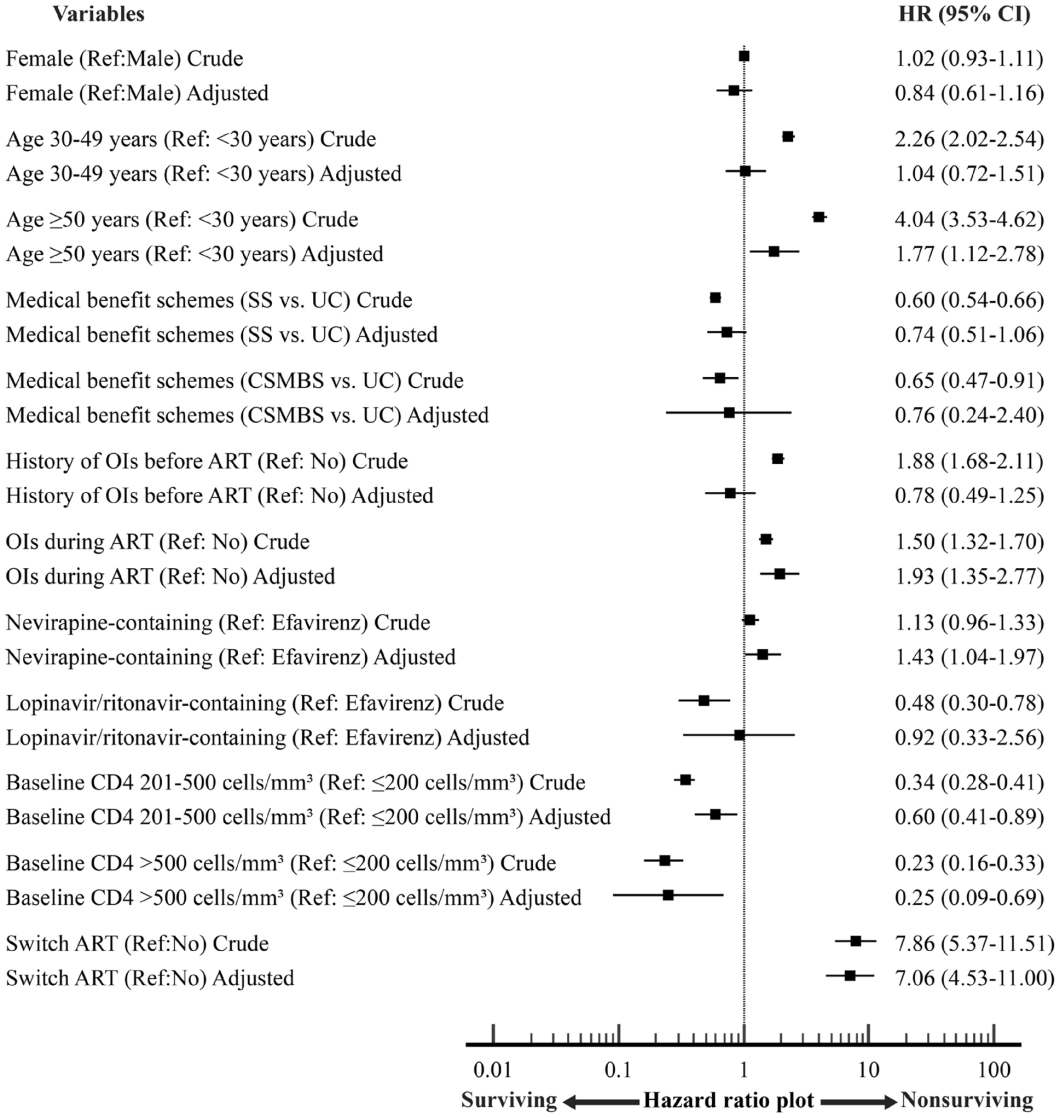
Hazard ratio plots of possible factors associated with risk of death

A total of 11,911 (85%) PLWH were alive during the follow-up period, and 2142 (15%) mortalities occurred in a total follow-up period of 28,254 patient-years. The mortality rate accounted for 7.5 (95% CI 7.2–7.9) per 100 person-years. The probability of survival after HIV registration estimated by the Kaplan–Meier method is shown in Fig. 2. Overall survival rates at 1, 2, 3, 4 and 5 years after HIV registration were 88.2% (95% CI 87.6–88.7%), 85.3% (95% CI 84.6–85.9%), 82.9% (95% CI 81.9–83.4%), 81.3% (95% CI 80.5–82.0%) and 75.1% (95% CI 73.5–76.8%), respectively. Forest plots of factors associated with the risk of death are shown in Fig. 3. The Cox proportional hazards model showed that all-cause mortality was associated with a history of ART switching (HR = 7.06, 95% CI 4.53–11.00), major opportunistic infections during ART (HR = 1.93, 95% CI 1.35–2.77), baseline CD4 count ≤ 200 vs. > 500 cells/mm3 (HR = 4.00, 95% CI 1.45–11.11), age ≥ 50 vs. < 30 years (HR = 1.77, 95%CI 1.12–2.78), and receiving nevirapine-based regimens (HR = 1.43, 95% CI 1.04–1.97). Figure 2 showed survival probability over 5-year study period of all patients (Fig. 2A) and were stratified by significant factors (Fig. 2B–F). The other factors were not associated with all-cause mortality. The most frequent reasons for ART switching included ART-related complications (52%).

## Discussion

This study estimated the survival rate of PLWH at a recent period of time and related risk factors associated with death based on the national AIDS program database. The overall survival rate is relatively low when compared to our previous report that was conducted over a decade ago in a tertiary HIV referral center among patients coinfected with HIV and TB [7]. In contrast, several studies in Europe and America have reported substantially reduced mortality rates after the introduction of highly effective combined ART [8–14]. However, the magnitude of the decrease in mortality rate in these continents was higher than that in resource-limited countries and even changed sooner [15–17]. This finding may be explained by the majority of PLWH presenting relatively late with very low CD4 cell counts. Approximately 40% of all PLWH presented with CD4 counts < 100 cells/mm^3^ and 90% presented with CD4 counts < 500 cells/mm^3^. Although HIV prevalence is significantly declining in Thailand due to successful HIV prevention programs, with the rate decreasing by approximately 60% between 2010 and 2018 [1], early identification of all PLWH and prompt initiation of ART in these individuals are essential for diligent HIV care [18, 19], which could lead to dramatic reductions in the death rate. Delays in linkage to medical care are associated with a greater likelihood of progression of the disease by immunologic deterioration. Our findings suggest that additional research and interventions on best practices for HIV testing and linkage to care is important. Such multifaceted approach to the problem of linkage to care remains vital.

The WHO estimated that of the 40 million people living with HIV/AIDS in the world, approximately 2.8 million are 50 years and older [20]. The present study demonstrated that elderly PLWH were 1.8 times more likely to die than younger PLWH. There are many challenges for the management of aging PLWH. These include chronic comorbidities, such as cardiovascular disease, osteoporosis and fractures, renal disease, chronic neurological disease, malignancies, liver disease, and metabolic syndrome, as well as geriatric syndromes and frailty [21–28]. In addition, elderly PLWH are more likely to experience lower status disclosure, social isolation, depression, and adverse clinical outcomes. The principles developed in geriatric medicine should be applied to a large portion of HIV patients. Particular management guidelines and recommendations for the optimal health care of elderly PLWH are essential.

In the present study, PLWH with major OIs after ART initiation were approximately twice as likely to die than those who did not have major OIs. Although the etiology of OIs cannot be retrieved from the database, tuberculosis is the leading cause of morbidity and mortality among PLWH worldwide, including Thailand [29]. One major challenge in the reduction of opportunistic infections associated with HIV infection is the difficulty of diagnosing and treating a new HIV case early before immune deterioration. A significant proportion of newly diagnosed persons have already experienced substantial immune deterioration. Early ART has shown a reduction in morbidity and mortality, in addition to preventing the transmission of HIV infection. A second challenge is that not all newly diagnosed PLWH receive timely continuous HIV care and even start ART. A third challenge is that retention in care to achieve and maintain durable virologic suppression and to reduce the chance of HIV-related OIs remains challenging in the resource-limited setting. Not all persons treated for HIV achieve durable viral suppression. Furthermore, immune reconstitution syndromes associated with new OIs might occur after ART initiation [30]. On the other hand, this is a reason why previous opportunistic infection were included in the multivariate analysis. Ultimately, durable virologic suppression eliminates most but not all major OIs. Tuberculosis remains at a higher prevalence in persons with HIV regardless of immune status, particularly in high TB endemic regions. The implementation of effective HIV and TB collaboration service programs is needed. ART regimen factor which stratified as efavirenz-containing or nevirapine-containing or lopinavir/ritonavir-containing regimen was followed the guidelines for antiretroviral therapy in HIV-1 infected adults and adolescents 2014, Thailand [31]. Non-nucleoside reverse transcriptase inhibitor (NNRTI) is potent, proven long-term efficacy, less drug-drug interactions and low cost. Thai HIV treatment guideline recommended NNRTI-based ART as the first-line recommended regimen for treatment-naive HIV-infected patients in the country during this study period. Efavirenz-based ART has shown durability, good virologic response, once daily used and is co-formulated with tenofovir and emtricitabines. Thus, efavirenz is recommended as the third drug combined with NRTI backbone. Nevirapine is more toxic during the first three months of therapy [32].

There were a number of limitations in the present study. First, it is a retrospective study reviewing secondary data; hence, some details of patients’ clinical condition may be underestimated and missed, such as, details of ART complications, characteristics of major opportunistic infections, nutritional status, baseline and follow-up weights, and baseline and follow-up WHO clinical stage. Furthermore, data regarding adherence to ART is lacking. Almost all of the PLWH in this study were initiated with NNRTI-based ART, which is less durable than the current recommended ART with integrase inhibitor-based regimen, and better treatment outcomes can be achieved despite lower adherence [33]. Integrase inhibitor-based ART regimens replacing PI- and NNRTI-based regimens have been implemented in Thailand since early 2021. In addition, the route of HIV transmission was omitted, though it is known to have an impact on morbidity and mortality [34]. Ultimately, the definite causes of death could not be identified owing to unavailable information in the database. However, the real-world evidence from the present study represents substantial all-cause mortality in Thai PLWH, even if the availability and accessibility of ART has improved in the past two decades. The present finding would support policy-makers and health care providers in further managing treatment for PLWH.

## Conclusion

In summary, this study demonstrated the substantial mortality rate over 5 consecutive years of the follow-up period. Patients who had a history of ART switching, major opportunistic infections, a lower baseline CD4 count, an older age, and received nevirapine-based regimens had an increased risk of death. Further prospective interventional studies with the aim of decreasing morbidity and mortality are needed in Thai PLWH. Active early case finding and prompt initiation of ART, which durable and less long-term side effect regimens, as well as continuous HIV care are a cornerstone to improve survival.

## Data Availability

The datasets used and/or analyzed during the present study are available from the corresponding author upon reasonable request.
